# Hazardous Waste: Electronics, Lead, and Landfills

**DOI:** 10.1289/ehp.112-a734

**Published:** 2004-09

**Authors:** Valerie J. Brown

Ironically, some of our most advanced technologies, when discarded, may represent a rapidly expanding and sometimes unregulated exposure to a toxicant that plagued even the ancient Romans: lead. Almost all electronic devices contain lead, and such devices are proliferating—and becoming obsolete—at breathtaking speed. A University of Florida environmental engineer is researching the potential environmental fate of the lead found in electronics sent to landfills. In a report sponsored by the U.S. Environmental Protection Agency (EPA) and issued 15 July 2004, Timothy G. Townsend described his study of 12 different types of electronic items and his finding that the items leached lead at concentrations exceeding the EPA threshold for categorizing a waste as hazardous.

Townsend’s goal is to help landfill regulators and managers decide how to allocate scarce resources. He explains, “Maybe they have to choose what type of waste to recycle—tires or electronics?” By discovering whether electronics leach toxic chemicals, he says, “we might help a community decide.” He focused on testing for lead because it happens to extract well under the test procedure he used—which is modeled on landfill conditions—and thus may be likely to leach from a landfill.

Townsend’s report, *RCRA Toxicity Characterization of Computer CPUs and Other Discarded Electronic Devices,* expanded on his earlier research on cathode ray tubes (CRTs) used in computer monitors and televisions. CRTs contain an average of about four pounds of lead. There are smaller quantities in the solder used in other electronic devices.

Townsend performed an EPA test known as the toxicity characteristic leaching procedure (TCLP) on a variety of electronic items including computer CPUs (central processing units), televisions, videocassette recorders, printers, cellular phones, remote controls, computer mice and keyboards, and smoke alarms. The TCLP test determines the mobility of analytes present in waste. Following the protocol, the devices were ground up, mixed with an acetic acid–based simulated leachate fluid, and rotated in a drum container for 18 hours, after which the leachate was tested for metal concentrations. In the TCLP, lead concentrations above 5 milligrams per liter are considered hazardous. All the devices Townsend tested leached lead over this threshold under some conditions.

But is the lead that is actually in landfills a health threat? “It has never been shown that lead is actually leaching out of landfills,” says Fern Abrams, director of environmental policy at IPC–Association Connecting Electronics Industries, an industry group based in Northbrook, Illinois. And although lead is known to be present in landfills, some of it may come from other constituents. “Electronics in general are one percent of the waste that goes into a landfill,” says Jan Whitworth, a policy analyst with the Oregon Department of Environmental Quality. So if lead were to be found in leachate, it would be very hard to say for sure whether it had come from electronics.

Even so, the European Union has banned lead solder in certain electronic devices beginning in 2006, due to landfill concerns. California already bans disposal of CRTs and televisions in household waste landfills. Oladele Ogunseitan, an associate professor of social ecology at the University of California, Irvine, who is evaluating the phaseout of lead solder, thinks it makes sense to allow manufacturers to use hazardous materials when alternatives are not available, but to require recycling. Today, many computer manufacturers will recycle discarded computers, but often will charge a fee.

Others believe hazardous substances must be removed from products altogether. Mamta Khanna, pollution prevention program manager at the nonprofit activist Center for Environmental Health in Oakland, California, would like electronics manufacturers to take cradle-to-grave responsibility for their products. “Once they have to bear the burden of disposal, they will use less hazardous materials,” says Khanna. “Why wait for years of study to determine when these toxic materials will start leaching and poisoning us, when electronics makers can start using safer materials today?” Khanna also points out that electronics waste is associated with other potentially toxic chemicals, including mercury, chromium, and brominated flame retardants.

To simulate landfill conditions more accurately than can be done in a lab with the TCLP, Townsend is now conducting an experiment in which he has buried garbage and electronics waste. Simulated rainfall is added periodically, with leachate forming as the water percolates through the waste. Results will be available in about two years. Next year the EPA expects to issue a rule limiting how CRTs can be disposed of nationwide, according to agency environmental protection specialist Marilyn Goode.

## Figures and Tables

**Figure:**
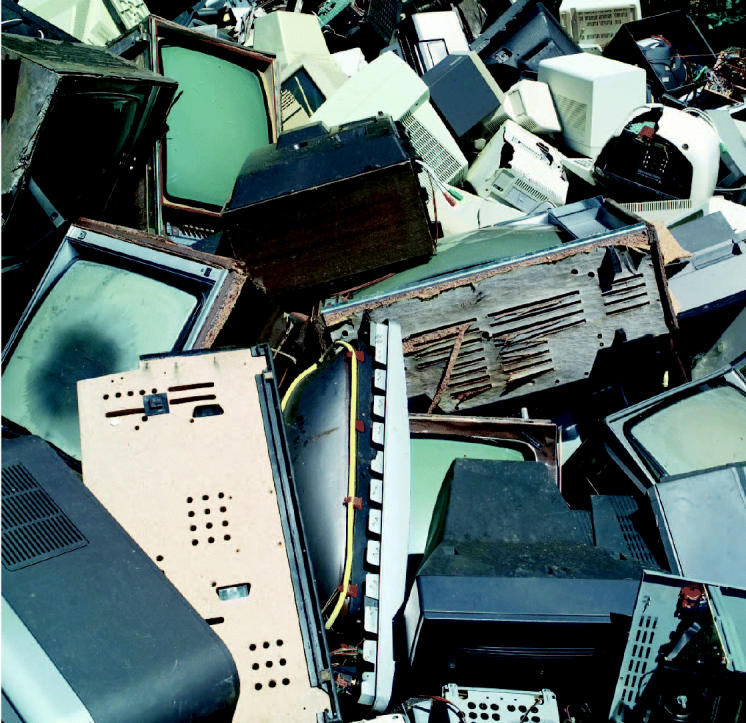
**Obsolete and overflowing.** Certain electronic items have become practically disposable, and are tossed into landfills as soon as the newest version arrives. Once there, however, are they leaching lead at hazardous rates?

